# Effects of surgery on cancer metastasis: biological mechanisms and perioperative implications

**DOI:** 10.3389/fonc.2026.1796434

**Published:** 2026-06-18

**Authors:** Mwangala Nalisa, Thifhelimbilu Emmanuel Luvhengo, Petrina Kapewangolo

**Affiliations:** 1 Department of Biochemistry, Microbiology and Biotechnology, Faculty of Agriculture and Natural Sciences, University of Namibia, Windhoek, Namibia; 2 Department of Surgery, University of the Witwatersrand, Johannesburg, South Africa

**Keywords:** cancer surgery, circulating tumor cells, immunotherapy, metastasis, perioperative immune suppression, perioperative oncology, prehabilitation, tumor microenvironment

## Abstract

Cancer metastasis remains the leading cause of cancer-related mortality, and the perioperative period has emerged as a critical window during which metastatic progression may be influenced. While surgical resection remains central to curative cancer treatment, accumulating preclinical, translational, and clinical evidence suggests that surgery-associated tissue injury, inflammation, neuroendocrine stress responses, immune perturbation, and host physiological factors can modulate metastatic dynamics in context-dependent ways. This review integrates experimental and clinical literature to examine the biological mechanisms through which surgery may influence metastatic progression, with emphasis on perioperative inflammatory responses, immune suppression, circulating tumor cells (CTCs), epithelial-mesenchymal transition (EMT), tumor dormancy, neutrophil extracellular traps (NETs), circulating tumor cell clusters, and emerging interactions involving the gut microbiome and tumor microenvironment. We additionally examine how perioperative physiological status, prehabilitation, and multidisciplinary optimization strategies may influence perioperative resilience and postoperative recovery. We further discuss emerging approaches aimed at mitigating surgery-associated metastatic vulnerability, including perioperative systemic therapies, immunomodulation, neoadjuvant and perioperative immunotherapy, minimally invasive surgical approaches, and tumor microenvironment targeted interventions. A clearer understanding of perioperative biological perturbations may inform the development of integrated perioperative oncology strategies to reduce metastatic risk and improve long-term oncologic outcomes.

## 1 Introduction

Surgical resection remains the primary curative modality for most solid malignancies in adults, aiming to achieve durable local control and prevent metastatic spread. However, growing evidence indicates that the perioperative period represents a biologically dynamic and potentially vulnerable window, characterized by tissue injury, systemic inflammation, neuroendocrine stress responses, and transient immune suppression, which may influence metastatic progression (1, 2).

Over the past two decades, advances in tumor biology, immunology, and perioperative medicine have reshaped understanding of how surgical interventions interact with metastatic processes. Experimental models, clinical recurrence analyses, and translational studies suggest that surgery can exert both tumor-inhibitory and tumor-promoting effects in a context-dependent manner (3–5).

This review synthesizes current mechanistic and clinical evidence on the effects of surgery on cancer metastasis, focusing on perioperative inflammatory and immunological alterations, circulating tumor cells, epithelial–mesenchymal transition (EMT), tumor dormancy, and emerging mechanisms including neutrophil extracellular traps (NETs), circulating tumor cell clusters, and surgery-associated alterations in the tumor microenvironment and host physiology. In addition, we discuss evolving perioperative strategies aimed at mitigating metastatic risk, including prehabilitation and physiological optimization, perioperative systemic therapy, immunomodulation, minimally invasive surgical approaches, and neoadjuvant immunotherapeutic interventions. By integrating molecular insights with clinical observations and contemporary perioperative oncology concepts, this review highlights opportunities to optimize surgical cancer care and improve long-term oncologic outcomes.

## 2 Mechanisms linking surgery to metastasis

Surgical resection, although essential for tumor control, induces perioperative biological changes that may inadvertently facilitate metastatic progression. Tissue injury associated with surgery triggers systemic inflammation, transient immune suppression, and tumor cell stress responses that can enhance tumor cell dissemination and outgrowth. This section reviews the principal mechanisms linking surgery to metastasis, with emphasis on perioperative inflammatory and immunological alterations, the release and survival of circulating tumor cells (CTCs) and micrometastases, and the activation of EMT programs that promote invasiveness and metastatic competence.

### 2.1 Inflammatory response and immune suppression

Surgery induces a systemic inflammatory response characterized by the release of cytokines such as interleukin-6 (IL-6), tumor necrosis factor-α (TNF-α), and vascular endothelial growth factor (VEGF), which can promote tumor cell survival, angiogenesis, and metastatic potential (6). Surgical stress concurrently triggers a transient but profound state of immunosuppression, marked by impairments in both innate and adaptive immunity. Clinical and experimental studies consistently demonstrate a postoperative reduction in natural killer (NK) cell cytotoxicity and CD8^+^ cytotoxic T-cell activity, key mediators of tumor immune surveillance, thereby facilitating the survival and outgrowth of CTCs during the perioperative period (7–9).

The perioperative period is further shaped by tissue injury induced wound-healing responses and psychological and physiological stress, which together create a systemic inflammatory and neuroendocrine milieu conducive to metastatic progression (1, 9). Surgical trauma increases the release of growth factors, cytokines, and pro-angiogenic mediators, while stress-induced elevations in catecholamines and prostaglandins suppress cell-mediated immunity, particularly NK-cell activity (1, 9). The convergent perioperative alterations promote tumor cell survival, invasion, angiogenesis, and dissemination, thereby facilitating the establishment and/or growth of pre-existing micrometastases and increasing the risk of postoperative cancer recurrence (1, 9).

Surgical tissue injury also results in the release of damage-associated molecular patterns (DAMPs), including high mobility group box 1 (HMGB1), extracellular ATP, heat shock proteins, and S100 proteins, which act as endogenous danger signals that amplify perioperative inflammation. These molecules activate pattern-recognition receptors such as Toll-like receptors (TLRs) and receptor for advanced glycation end products (RAGE), triggering NF-κB–dependent inflammatory signaling and inflammasome activation (10, 11). In the postoperative setting, persistent DAMP signaling may contribute not only to tissue repair responses but also to tumor-promoting inflammation, enhanced vascular permeability, and recruitment of immunosuppressive myeloid populations that facilitate metastatic progression.

Beyond transient perioperative suppression of NK-cell and cytotoxic T-cell activity, surgical trauma induces a more sustained immunosuppressive state characterized by expansion of regulatory T cells (Tregs), myeloid-derived suppressor cells (MDSCs), and alternatively activated macrophages (4, 12, 13). MDSCs are increasingly recognized as central mediators of postoperative immune dysfunction, as they suppress T-cell activation through arginase-1 activity, reactive oxygen species production, nitric oxide signaling, and secretion of immunosuppressive cytokines including IL-10 and TGF-β (14, 15). Concurrent Treg expansion further inhibits antitumor immunity by impairing dendritic cell maturation and suppressing effector T-cell proliferation and cytokine production. Importantly, postoperative expansion of MDSC and Treg populations may persist beyond the immediate perioperative period, extending the period of immune vulnerability beyond the immediate perioperative phase and creating a sustained window permissive for survival and metastatic outgrowth of residual malignant cells (4, 9).

Postoperative inflammatory mediators further contribute to sustained immune dysfunction by perturbing metabolic, neuroendocrine, and immune signaling pathways essential for effective immune activation. In particular, surgical trauma induces cyclooxygenase-2–dependent prostaglandin E_2_ (PGE_2_) production, which suppresses NK-cell cytotoxicity and T-cell proliferation while reinforcing pro-inflammatory signaling cascades (4, 12, 13). Concurrent activation of damage-associated inflammatory pathways and acute-phase responses further disrupts lymphocyte activation and cellular immune surveillance. Collectively, perioperative inflammatory signaling, DAMP-mediated immune activation, and prolonged expansion of immunosuppressive myeloid and regulatory lymphocyte populations establish a systemic environment that may favor metastatic seeding, immune evasion, and postoperative tumor recurrence.

In addition to systemic inflammatory and immunological alterations, growing evidence suggests that perioperative disruption of the gut microbiome may further contribute to postoperative immune dysregulation and metastatic susceptibility.

### 2.2 Microbiome dysbiosis and surgical recurrence

Increasing evidence suggests that surgical interventions can induce clinically significant alterations in the gut microbiome, with potential implications for postoperative inflammation, mucosal healing, immune regulation, and cancer recurrence. Gastrointestinal surgery in particular disrupts microbial homeostasis through tissue manipulation, ischemia–reperfusion injury, bowel preparation, perioperative antibiotics, altered nutrient availability, and changes in intestinal oxygenation. These perioperative perturbations may promote microbial dysbiosis characterized by reduced microbial diversity, depletion of commensal bacteria, and expansion of pro-inflammatory or pathogenic taxa (16, 17).

Emerging data further indicate that different colorectal surgical procedures may induce distinct postoperative microbial alterations due to the inherent regional variation of the colonic microbiome. The right and left colon differ in luminal oxygen tension, bile acid exposure, nutrient availability, transit time, and microbial composition, resulting in region-specific microbial ecosystems and metabolic profiles (18, 19). Surgical resection of different colonic segments may therefore differentially influence microbial diversity, short-chain fatty acid production, mucosal immune regulation, and epithelial barrier integrity (19). These postoperative microbial shifts may contribute to variations in inflammatory responses, mucosal healing, and susceptibility to tumor-promoting immune dysregulation following colorectal surgery (18, 19). Clinical studies have reported location-associated differences in disease-free survival and recurrence outcomes between right- and left-sided colorectal cancers; however, findings remain inconsistent and the contribution of postoperative microbiome alterations to these differences has not been established (20).

Postoperative dysbiosis may contribute to metastatic progression through several interconnected mechanisms. Disruption of epithelial barrier integrity can facilitate bacterial translocation and sustained inflammatory activation, while enrichment of pathogenic bacteria may enhance production of pro-inflammatory cytokines, reactive oxygen species, and tumor-promoting metabolites. Certain microbial populations have also been implicated in modulation of inflammatory and immunosuppressive pathways, including recruitment of myeloid-derived suppressor cells (MDSCs), regulatory T-cell expansion, angiogenesis, and suppression of antitumor immune responses, thereby creating a microenvironment permissive for metastatic establishment (18, 19). In addition, impaired microbiome recovery following surgery may negatively influence wound healing and anastomotic integrity, while persistent postoperative dysbiosis may contribute to ongoing inflammatory signaling and immune dysfunction (17, 21).

Although direct evidence linking surgery-induced microbiome alterations to metastatic recurrence remains limited, growing translational and clinical evidence supports the microbiome as a potentially important mediator of perioperative oncologic outcomes. Future studies integrating longitudinal microbiome profiling, immune phenotyping, and clinical recurrence data will be essential to clarify causal relationships and identify microbiome-targeted perioperative interventions aimed at reducing postoperative metastatic risk.

### 2.3 Circulating tumor cells and micrometastases

Circulating tumor cells, malignant cells that detach from the primary tumor and enter the bloodstream, are key intermediates in hematogenous metastasis and are strongly associated with disease recurrence and reduced survival across multiple cancer types (22). Although CTCs may be released spontaneously during tumor progression, accumulating evidence indicates that surgical manipulation of tumors can acutely increase CTC mobilization into the circulation (5).

Clinical studies have demonstrated that surgical intervention can be associated with transient perioperative increases in CTCs, suggesting that tumor manipulation may contribute to tumor cell dissemination (23, 24). In non-small-cell lung cancer, intraoperative sampling of pulmonary venous blood revealed a significant rise in CTC counts following surgical manipulation of the tumor, with the magnitude of increase particularly pronounced in patients exhibiting microscopic lymphatic invasion, supporting a direct role for mechanical disruption of tumor and vascular structures in the release of CTCs (23). Similar perioperative dynamics have been reported in colorectal cancer, where CTCs were detected during and immediately after hepatic resection for liver metastases, and their appearance in peripheral blood was attributed to the surgical procedure itself, indicating that tumor handling can transiently promote entry of tumor cells into the systemic circulation (24). In breast cancer, while CTCs detection in the immediate postoperative period has not consistently demonstrated prognostic significance, the presence of CTCs before surgery and during longitudinal postoperative follow-up has been associated with increased risk of recurrence and reduced disease-free survival, underscoring the clinical relevance of persistent or re-emerging CTCs rather than short-lived postoperative fluctuations (25).

Once in the circulation, CTCs may exist as single cells or as multicellular clusters, the latter demonstrating substantially greater metastatic potential due to enhanced resistance to shear stress and immune-mediated destruction (26). Many CTCs exhibit EMT phenotypes, conferring increased invasiveness and stem-like properties that promote colonization of distant organs (22).

Surgery may influence the behavior of micrometastases. Micrometases are small, clinically undetectable tumor cell deposits at secondary sites. Evidence from patients with breast cancer suggests that surgical removal of the primary tumor can alter systemic angiogenic and inflammatory signaling, potentially reactivating dormant micrometastatic foci and promoting their progression to overt metastases (27). Advances in molecular detection methods, including reverse transcription-PCR (rt-PCR) and circulating tumor DNA analyses, have improved identification of minimal residual disease, underscoring the clinical relevance of perioperative tumor cell dissemination (22).

Collectively, the above findings highlight a paradox of cancer surgery. While surgical resection is essential for curative intent, the perioperative period may transiently enhance metastatic risk through increased mobilization of CTCs and activation or outgrowth of micrometastatic disease (5, 28). Recognition of this period of increased vulnerability to metastasis has led to a growing interest in perioperative strategies aimed at minimizing tumor cell dissemination and metastatic progression, including refinement of surgical techniques such as non-touch or minimally invasive approaches, preservation or modulation of perioperative immune function, and systemic perioperative interventions designed to counteract inflammation- and stress-mediated promotion of survival of CTCs and metastatic seeding (1, 4, 5).

### 2.4 Epithelial mesenchymal transition

Epithelial-mesenchymal transition is a conserved biological program in which epithelial tumor cells lose apico-basal polarity and intercellular adhesion while acquiring mesenchymal traits associated with increased motility, invasiveness, and resistance to apoptosis (29). Epithelial–mesenchymal transition is a well-established driver of metastatic dissemination in solid tumors, enabling cancer cells to invade surrounding tissue, intravasate into the circulation, survive systemic transport, and ultimately colonize distant organs (29, 30).

During oncologic surgery, experimental evidence demonstrates that tissue injury initiates a wound-healing response characterized by the release of inflammatory cytokines and growth factors, including transforming growth factor-β (TGF-β), which have been shown to activate EMT-associated transcriptional programs in residual tumor cells (31). In murine models, invasive procedures such as biopsy or partial tumor resection trigger wound-healing-like inflammatory responses characterized by increased expression of EMT associated transcription factors and mesenchymal markers, accompanied by enhanced tumor cell migration, local invasion, and metastatic dissemination (31).

At the molecular level, TGF-β which is rapidly upregulated during tissue repair following surgery, is a central inducer of EMT (2, 31). Additionally, TGF-β signaling suppresses epithelial markers such as E-cadherin while inducing mesenchymal markers including vimentin and N-cadherin through activation of transcription factors such as Snail, Slug, and ZEB family proteins (31). Concurrently, inflammation induced by surgery amplifies EMT signaling through cytokines such as IL-6 and TNF-α, which activate STAT3 and NF-κB pathways, reinforcing EMT and promoting tumor cell survival and motility (2, 5). These key EMT regulators and their surgery-associated metastatic roles are summarized in **Table 1**.

**Table 1:** Key EMT regulators and metastatic niche regulators implicated in surgery-associated metastatic progression.

EMT regulator	Source during surgery	EMT-related effects	Evidence in surgery/metastasis	Key references
TGF-β	Released during tissue injury and wound-healing responses following biopsy or tumor resection	Represses epithelial markers (e.g., E-cadherin); induces mesenchymal markers (vimentin, N-cadherin); activates EMT transcription factors (Snail, Slug, ZEB)	Biopsy and surgical resection induce TGF-β upregulation and EMT-associated transcriptional changes that promote invasion and metastatic outgrowth	Alieva et al. (31); Lamouille et al. (30); Tohme et al. (5)
Snail/Slug	Induced downstream of TGF-β and inflammatory signaling triggered by surgical injury	Repress epithelial gene expression; enhance tumor cell motility and invasion	Increased EMT-related transcriptional activity and invasive behavior observed following biopsy or partial tumor resection in preclinical models	Alieva et al. (31); Kalluri and Weinberg (29)
ZEB1/ZEB2	Activated by TGF-β, inflammatory cytokines, hypoxia, and perioperative stress signaling	Promote EMT plasticity, stemness, immune evasion, and metastatic dissemination	Emerging evidence indicates that inflammatory and hypoxic perioperative conditions may enhance ZEB-mediated EMT programs associated with metastatic progression and therapy resistance	Lamouille et al. (30); Dongre and Weinberg (82)
IL-6	Produced during postoperative inflammatory responses	Activates STAT3 signaling; promotes EMT-associated survival, migration, and invasion	Surgery-induced inflammation elevates IL-6/STAT3 signaling, enhancing tumor cell survival, invasion, and metastatic establishment in the perioperative period	Hiller et al. (2); Tohme et al. (5)
TNF-α	Acute inflammatory cytokine released after surgical trauma	Activates NF-κB signaling; cooperates with TGF-β to sustain EMT programs	Surgical trauma induces TNF-α–dependent inflammatory signaling that promotes tumor cell adhesion, invasion, and metastatic efficiency	Kalluri and Weinberg (29); Tohme et al. (5)
Matrix metalloproteinases (MMP-2, MMP-9)	Upregulated during wound repair, NET-associated inflammation, and postoperative stromal remodeling	Degrade extracellular matrix; promote EMT, endothelial invasion, angiogenesis, and metastatic niche remodeling	Surgical inflammation and tissue remodeling increase MMP activity, facilitating extracellular matrix degradation, tumor cell invasion, vascular permeability, and establishment of a permissive postoperative metastatic niche	Kalluri and Weinberg (29); Tohme et al. (5); Zhang et al. (83)
CD36/fatty acid oxidation pathways	Induced by inflammatory and metabolic stress within the postoperative microenvironment	Enhance metabolic plasticity, survival of disseminated tumor cells, and metastatic niche colonization	Emerging evidence suggests surgery-associated inflammatory signalling and NET formation may promote lipid metabolic adaptation and FAO-dependent survival in postoperative metastatic niches	Haykal et al. (32); Pascual et al. (35)

ECM, extracellular matrix; EMT, epithelial–mesenchymal transition; FAO, fatty acid oxidation; IL-6, interleukin-6; MMPs, matrix metalloproteinases; NETs, neutrophil extracellular traps; NF-κB, nuclear factor kappa B; Snail/Slug, SNAI family zinc finger transcription factors; STAT3, signal transducer and activator of transcription 3; TGF-β, transforming growth factor beta; TNF-α, tumor necrosis factor alpha; ZEB, zinc finger E-box–binding homeobox.

Importantly, EMT induced during the perioperative period may cooperate with other surgery associated processes, including immune suppression and extracellular matrix remodeling, to enhance metastatic efficiency. Consequently, EMT-like tumor cells display increased resistance to immune-mediated killing and improved adaptability within distant organ microenvironments, facilitating metastatic colonization during the postoperative window (5, 29). Collectively, the above findings suggest that surgery-induced EMT represents a mechanistic link between operative stress, tumor cell plasticity, and increased metastatic risk.

From a translational perspective, although direct causal evidence for surgery-induced EMT in humans remains limited, postoperative inflammatory mediators and perioperative tissue-injury responses that are functionally consistent with EMT-like phenotypes have been associated with enhanced invasion, metastatic progression, and poorer oncologic outcomes (5). Taken together, the observations support that EMT represents a mechanistic link between surgical trauma, inflammation, and increased metastatic risk during the perioperative period.

### 2.5 Neutrophil-metabolism axis in surgery-associated metastasis

Emerging evidence indicates that the interaction between neutrophil-driven inflammation and tumor metabolic adaptation represents an important mechanism linking surgical stress to metastatic progression (32). Surgical trauma induces rapid activation of neutrophils and formation of neutrophil extracellular traps (NETs), web-like extracellular chromatin structures enriched with proteases, histones, and inflammatory mediators. Although NET formation is physiologically involved in antimicrobial defense and tissue repair, excessive perioperative NETosis has increasingly been implicated in postoperative metastatic progression. NETs may promote metastasis by trapping circulating tumor cells, enhancing tumor cell adhesion to vascular endothelium, and establishing a pro-inflammatory microenvironment that facilitates metastatic colonization (33, 34).

Recent studies further demonstrate that surgery-induced NETs contribute to metabolic reprogramming of disseminated tumor cells (DTCs). In particular, NET-associated inflammatory signaling has been linked to activation of MYC-dependent transcriptional programs and increased fatty acid oxidation (FAO), enabling tumor cells to survive oxidative stress and nutrient deprivation within the postoperative metastatic niche (32). Fatty acid uptake receptors, especially CD36, appear to play a central role in this adaptive response (35, 36). CD36-mediated lipid uptake promotes lipid metabolic reprogramming and has been linked to enhanced tumor growth, survival, and metastatic progression through improved utilization of fatty acids as an energy source (37, 38). Increased CD36 expression has also been associated with metastatic competence and adaptation to lipid-rich microenvironments (35, 36).

This emerging neutrophil-metabolism axis provides a mechanistic bridge between perioperative inflammation and metastatic outgrowth. Surgical stress-induced NETosis may therefore not only facilitate physical trapping and dissemination of tumor cells, but also promote metabolic adaptations that support survival and expansion of micrometastatic disease. Emerging evidence further suggests that NET-associated inflammatory signaling may contribute to reactivation of dormant disseminated tumor cells. NET-derived proteases can remodel extracellular matrix components, particularly laminin, promoting awakening of previously quiescent tumor cells and thereby providing a potential mechanistic link between surgery-associated inflammation and dormancy escape (39). These findings further support investigation of perioperative strategies targeting NET formation, neutrophil activation, and tumor lipid metabolism as potential approaches to reduce surgery-associated metastatic risk.

## 3 Clinical evidence linking surgery to metastatic risk

Beyond mechanistic insights, a growing body of clinical evidence suggests that surgical intervention may influence metastatic risk in context-dependent ways. Long-term follow-up and recurrence hazard analyses indicate that perioperative events may coincide with non-random temporal patterns of metastatic relapse, consistent with transient immune suppression, altered tumor dormancy dynamics, and tumor cell reprogramming toward more permissive or invasive phenotypic states. Conversely, surgical resection can reduce tumor burden and eliminate sources of systemic tumor-promoting signals, supporting its anti-metastatic potential in selected clinical contexts. This section synthesizes clinical evidence for both the pro- and anti-metastatic effects of surgery, with particular emphasis on perioperative immune modulation, clinical patterns consistent with dormancy escape and phenotypic plasticity, and outcomes associated with cytoreductive and metastasis-directed surgical strategies.

### 3.1 Pro-metastatic effects

Clinical evidence accumulated over several decades indicates that surgical resection of primary tumors may, in some patients be associated with altered patterns of metastatic recurrence (3, 40, 41). This phenomenon has been most consistently described in malignancies characterized by high rates of distant relapse despite effective local disease control, such as breast cancer, and has also been reported in colorectal cancer (3, 41). While surgery remains essential for curative treatment of malignant solid tumors in adults, the perioperative period may coincide with an increased risk of metastatic progression, as reflected by non-random temporal clustering of distant relapse events following tumor resection in some patients (3, 40).

Long-term follow-up studies have demonstrated reproducible, non-linear recurrence hazard patterns following surgical treatment of breast cancer, characterized by early peaks in distant metastasis shortly after surgery (3, 40). Analyses of recurrence dynamics indicate that these early peaks are temporally associated with primary tumor removal rather than with subsequent adjuvant therapy, suggesting that factors temporally associated with surgical intervention may influence the timing of metastatic relapse (40). Such recurrence patterns have been observed across independent patient cohorts, supporting their robustness as a clinical phenomenon. The above clinical observations support the interpretation that surgical resection may influence the growth of pre-existing disseminated tumor cells, rather than acting solely by preventing further dissemination (3, 41). In particular, recurrence patterns characterized by abrupt transitions from prolonged latency to overt metastatic disease are consistent with clinical dormancy frameworks in which previously quiescent micrometastases are postulated to resume growth following primary tumor removal (3, 41). Importantly, these interpretations are derived from analyses of recurrence timing and hazard-rate patterns in patient populations, rather than from direct experimental manipulation.

Comparable temporal associations between surgical intervention and metastatic outcomes have been reported in other malignant solid tumors. In colorectal cancer cohorts, clinical studies have demonstrated that the persistent presence of CTCs in peripheral blood following curative-intent surgery is associated with significantly reduced disease-free and overall survival, indicating a higher risk of postoperative metastatic relapse (42). These findings suggest that the persistence of CTCs in the perioperative period may reflect survival of disseminated tumor cells contributing to adverse oncologic outcomes in a subset of patients, although direct causality cannot be definitively established from observational data alone.

When considered alongside clinical data demonstrating transient perioperative immune suppression (Section 3.1.1), these observations suggest that the postoperative period may represent a period of increased biological vulnerability, during which residual tumor cells may be less effectively constrained by the host’s immune defenses (43, 44). Furthermore, recurrence kinetics characterized by the sudden emergence of metastatic disease following extended disease-free intervals are consistent with clinical dormancy frameworks discussed in Section 3.1.2, in which previously quiescent disease is thought to resume growth, although such associations remain inferential in the absence of direct clinical biomarkers of dormancy escape (3, 41). Collectively, available clinical evidence supports an association between surgical intervention and pro-metastatic outcomes in selected contexts. Importantly, these observations do not challenge the necessity of surgery but rather underscore the importance of identifying perioperative risk modifiers and optimizing clinical strategies to mitigate unintended metastatic consequences (2, 3).

#### 3.1.1 Clinical evidence of perioperative immune suppression

Clinical studies indicate that surgical intervention is associated with a transient suppression of perioperative immune competence, particularly affecting innate immune surveillance mechanisms during the immediate postoperative period (43, 44). In patients undergoing major cancer surgery, postoperative reductions in NK-cell cytotoxicity have been documented within hours of surgery and may persist for several days, temporally overlapping with a period during which disseminated tumor cells may be less effectively constrained by the host’s defenses (43).

Perioperative immune suppression is associated with inferior oncologic outcomes, including increased risk of cancer recurrence and reduced survival following curative-intent surgery (44). Although immune parameters are not uniformly measured across studies, these associations support that perioperative immune dysfunction may act as a modifier of metastatic risk, particularly in patients harboring residual microscopic disease. Consistent with the recurrence-timing analyses discussed in Sections 3.1 and 3.1.2, these observations suggest that the postoperative period represents a biologically vulnerable period, during which transient reductions in immune constraint may permissively interact with dormant or disseminated tumor cells.

#### 3.1.2 Clinical evidence from anesthetic and perioperative intervention trials

Growing interest in perioperative modulation of metastatic risk has led to investigation of whether anesthetic technique influences long-term oncologic outcomes following cancer surgery. Experimental studies suggest that volatile anesthetics may promote perioperative immunosuppression, inflammation, hypoxia-inducible signaling, and tumor cell migration, whereas propofol-based total intravenous anesthesia (TIVA) has been associated with preservation of NK-cell activity, attenuation of inflammatory responses, and inhibition of tumor-promoting signaling pathways (45, 46). These observations have prompted considerable clinical interest in whether anesthetic selection may influence postoperative recurrence and survival outcomes.

Retrospective clinical studies and several meta-analyses have reported improved recurrence-free and overall survival in patients receiving propofol-based TIVA compared with volatile anesthetics, particularly in hepatocellular carcinoma, esophageal cancer, and certain gastrointestinal malignancies (45–47). Proposed mechanisms include reduced perioperative immune suppression, decreased inflammatory cytokine release, and preservation of cell-mediated antitumor immunity. However, the clinical evidence remains heterogeneous, with substantial variability in study design, cancer type, perioperative management, and duration of follow-up (45, 47).

More recent randomized clinical evidence has yielded less definitive conclusions. The VAPOR-C feasibility study and more recent comparative analyses have highlighted the challenges of establishing definitive oncologic superiority of either volatile or intravenous anesthetic techniques across diverse cancer populations (48). Similarly, recent 2025 meta-analyses comparing propofol-based TIVA with volatile anesthetics such as sevoflurane reported that although some subgroup analyses favored TIVA in selected malignancies, pooled evidence remained insufficient to establish definitive clinical superiority or support universal changes in anesthetic practice (45, 46). Importantly, many studies remain limited by retrospective design, confounding related to tumor stage and perioperative care, and heterogeneity in adjuvant oncologic treatment (45, 46).

Current evidence therefore suggests that anesthetic technique may influence perioperative tumor biology in specific clinical contexts, but a clear consensus regarding its impact on long-term metastatic recurrence has not yet been achieved. Future biomarker-driven and cancer-specific randomized trials incorporating immune profiling, circulating tumor cell dynamics, and standardized perioperative protocols will be essential to determine whether anesthetic modulation can meaningfully improve oncologic outcomes following cancer surgery (45, 46, 48).

A growing body of evidence further suggests that perioperative oncologic outcomes are influenced not only by anesthetic and immunomodulatory factors, but also by the urgency of surgical intervention and the patient’s underlying physiological reserve at the time of surgery.

#### 3.1.3 Influence of surgical urgency and preoperative physiological status

Clinical evidence further suggests that the metastatic and postoperative consequences of surgery may differ substantially between planned elective oncologic procedures and urgent or emergency surgical interventions. Patients undergoing urgent surgery for advanced or metastatic malignancy often present with significant physiological derangements, including systemic inflammation, malnutrition, anemia, hypoalbuminemia, impaired functional status, and pre-existing immune dysfunction. These factors may amplify perioperative stress responses and reduce physiological reserve, thereby increasing susceptibility to postoperative complications, immune suppression, and adverse oncologic outcomes (49–52).

This distinction is particularly evident in metastatic spine disease, where patients requiring urgent decompression or stabilization procedures demonstrate significantly higher perioperative morbidity, postoperative mortality, and failure-to-rescue rates compared with patients undergoing more carefully optimized elective interventions (51–53). In many cases, emergent surgical intervention limits opportunities for correction of reversible physiological abnormalities such as anemia, hypoalbuminemia, electrolyte imbalance, and systemic inflammatory burden prior to surgery. These preoperative factors are independently associated with impaired wound healing, infectious complications, prolonged hospitalization, and reduced overall survival, with hypoalbuminemia increasingly recognized as a clinically important marker of perioperative vulnerability and failure-to-rescue risk in metastatic spine surgery (52, 53).

From a mechanistic perspective, urgent surgical intervention may intensify perioperative inflammatory and neuroendocrine responses already heightened by advanced systemic disease, thereby potentially exacerbating postoperative immune dysfunction and creating a physiological environment permissive for adverse oncologic outcomes (49, 50). Conversely, elective oncologic pathways may allow implementation of prehabilitation strategies, nutritional optimization, correction of metabolic abnormalities, and multidisciplinary perioperative planning aimed at improving physiological resilience and postoperative recovery (49, 50).

These observations reinforce the importance of the preoperative optimization phase as a critical component of the oncologic surgical journey. Future studies should further investigate how perioperative physiological reserve, frailty, nutritional status, and inflammatory biomarkers interact with surgery-associated cancer biology and whether targeted preoperative interventions can improve both surgical and long-term oncologic outcomes. These findings further support the concept that perioperative oncologic outcomes are influenced not only by tumor biology and surgical factors, but also by the patient’s underlying physiological and immunological resilience at the time of intervention.

#### 3.1.4 Clinical evidence consistent with dormancy escape and phenotypic plasticity

Although direct clinical measurement of tumor dormancy and phenotypic transition remains challenging, patterns of metastatic recurrence observed in long-term follow-up studies of patients following cancer surgery are consistent with dormancy-based clinical models. For example, in patients with breast cancer, recurrence hazard analyses reveal prolonged periods of metastatic quiescence followed by sudden increases in relapse incidence, suggesting transitions from dormant to active disease states (3, 41). The aforementioned clinical pattern is compatible with frameworks proposing phenotypic plasticity and dormancy escape, although causality cannot be directly inferred from patient data alone. Importantly, the interpretations rely on population-level recurrence kinetics rather than experimental demonstration of EMT or dormancy disruption in individual patients. As such, clinical evidence supports an associative, rather than mechanistic link between surgery and phenotypic changes in disseminated tumor cells (3, 41).

### 3.2 Anti-metastatic effects of surgery

While surgery has traditionally been viewed as a local control strategy, accumulating clinical evidence indicates that surgical intervention can also confer survival benefits consistent with reduced metastatic progression in selected oncologic contexts. Across multiple cancer types, complete or near-complete resection of primary tumors and/or metastatic deposits has been associated with prolonged survival, particularly in patients with limited metastatic burden, favorable tumor biology, and adequate physiological reserve (54–56). These benefits are most evident when surgery is integrated with systemic therapies and applied within the framework of cytoreduction or metastasis-directed treatment (57, 58). Importantly, the survival benefits summarized below should be interpreted within the context of modern multidisciplinary oncology, where careful patient selection, molecular profiling, response to systemic therapy, and integration of surgery with chemotherapy, targeted therapy, and immunotherapy increasingly guide treatment decisions.

**Table 2** summarizes representative malignancies in which surgical intervention has been associated with survival benefit in selected metastatic settings, outlining the surgical approach, the nature of the reported benefit, the corresponding level of supporting evidence, and the evolving role of surgery within the modern multimodal and biomarker-driven treatment era. Collectively, these examples underscore that the oncologic impact of surgery in metastatic disease is highly context-dependent and increasingly linked to disease biology, systemic therapy responsiveness, multidisciplinary treatment planning, and appropriate patient selection, rather than reflecting a universal benefit of surgical intervention alone.

**Table 2:** Surgical intervention and survival outcomes in selected metastatic disease settings in the modern multimodal treatment era.

Cancer type	Surgical approach	Reported survival benefit	Level of evidence [Burns et al. (84)]	Role in the modern multimodal and biomarker-driven era	Key references
Colorectal cancer (CRC)	Resection of primary tumor ± metastasectomy (liver, lung, peritoneum)	Long-term survival and, in a subset of patients, potential cure following complete (R0) metastasectomy or cytoreductive surgery, particularly in oligometastatic disease with favorable biology	Level II	Increasingly integrated with perioperative chemotherapy, biologic agents, ctDNA-guided surveillance, and molecularly informed patient selection; outcomes are strongly influenced by response to systemic therapy and oligometastatic disease biology	Pfannschmidt et al. (85); Verwaal et al. (86); Adam et al. (87); Nordlinger et al. (68)
Renal cell carcinoma (RCC)	Cytoreductive nephrectomy ± metastasectomy	Improved overall survival in carefully selected metastatic patients, particularly in good-risk disease and in the pre-targeted therapy era; benefit is not universal and is refined by modern systemic therapy trials	Level I (context-dependent)	The role of cytoreductive nephrectomy has evolved substantially in the era of immune checkpoint inhibitors and VEGF-targeted therapies, with surgery increasingly reserved for carefully selected responders or low-burden metastatic disease	Flanigan et al. (54); Mickisch et al. (88), refined by Méjean et al. (89); Motzer et al. (90)
Ovarian cancer	Primary or interval cytoreductive (debulking) surgery	Residual disease volume is a dominant predictor of overall survival; complete cytoreduction (no macroscopic residual disease) confers the longest survival, irrespective of timing of surgery	Level I	Cytoreductive surgery is now closely integrated with platinum-based chemotherapy, bevacizumab, and maintenance PARP inhibitors, with survival increasingly influenced by molecular biomarkers including BRCA and homologous recombination deficiency status	Bois et al. (91); Bristow (55); Vergote et al. (92); Moore et al. (93)
Breast cancer (oligometastatic/*de novo* stage IV)	Resection of primary tumor and/or isolated metastases	Prolonged progression-free and overall survival in selected patients, with benefit dependent on tumor subtype, metastatic burden, and patient characteristics	Level I	Surgical benefit is increasingly interpreted within the context of HER2-targeted therapy, endocrine therapy, CDK4/6 inhibitors, and systemic disease control achieved with modern precision oncology approaches	Badwe et al. (94); Soran et al. (58); Finn et al. (95); Swain et al. (96)
Melanoma	Metastasectomy (lung, gastrointestinal tract, brain, soft tissue)	Durable long-term survival following complete resection in carefully selected patients, including in the era of modern systemic therapies	Level IV	Metastasectomy is increasingly combined with immune checkpoint inhibitors and BRAF/MEK-targeted therapies, substantially improving long-term disease control in selected patients	Hanna et al. (97); Nelson et al. (98); Ollila et al. (99); Robert et al. (100); Long et al. (101)
Soft-tissue sarcoma/osteosarcoma	Pulmonary metastasectomy	Significantly improved long-term survival after complete lung metastasectomy, particularly in patients with limited metastatic burden and favorable prognostic features	Level IV	Surgery remains central for selected patients with pulmonary oligometastatic disease but is increasingly integrated with systemic chemotherapy, individualized risk assessment, and multidisciplinary sarcoma management pathways	Chudgar et al. (102); Pastorino et al. (56); Gronchi et al. (103)
Neuroendocrine tumors (hepatic metastases)	Hepatic metastasectomy and/or cytoreductive surgery	Surgical resection or cytoreduction of liver metastases is associated with prolonged overall survival and improved symptom control in carefully selected patients, including when complete (R0) resection is not feasible	Level IV	Surgical cytoreduction is increasingly combined with somatostatin analogues, peptide receptor radionuclide therapy (PRRT), targeted agents, and liver-directed therapies	Howe et al. (104); Mayo et al. (105); Sarmiento et al. (106); Strosberg et al. (107)
Oligometastatic non-small-cell lung cancer	Local consolidative therapy (surgery and/or radiotherapy) following systemic therapy	Addition of metastasis-directed local therapy is associated with significantly improved progression-free survival and favorable overall survival trends, compared with systemic therapy alone, in selected patients without disease progression after first-line treatment	Level I	Local consolidative surgery is increasingly integrated with contemporary systemic treatment strategies including targeted therapy and immunotherapy with treatment selection guided by disease burden, treatment response, and molecular profiling in selected patients	Gomez et al. (57); Patchell et al. (108); Forde et al. (80); Lu et al. (81)
Gastric cancer (oligometastatic/limited metastatic disease)	Gastrectomy with metastasectomy or cytoreductive surgery after response to systemic therapy	Potential survival benefit reported in highly selected patients with oligometastatic disease who achieve complete (R0) resection following systemic therapy; no overall survival benefit with upfront cytoreductive gastrectomy, and surgical intervention remains investigational	Level I–II (exploratory)	Surgical intervention is increasingly considered after favorable response to systemic chemotherapy and emerging immunotherapy-based regimens, with patient selection guided by disease biology and response assessment	Goetze and Al-Batran (109); Hara et al. (110); Fujitani et al. (111); Janjigian et al. (112)
Pancreatic neuroendocrine tumors (metastatic)	Primary tumor resection ± liver metastasectomy or cytoreductive surgery	Surgical resection of the primary tumor and/or liver metastases is associated with improved long-term survival and symptom control in carefully selected metastatic patients, including in some cases with incomplete cytoreduction	Level IV	Surgical management is increasingly individualized within multimodal strategies incorporating PRRT, targeted therapy, somatostatin analogues, and liver-directed approaches	Partelli et al. (113); Howe et al. (104); Pavel et al. (114)

Levels of evidence were classified according to the hierarchy described by Burns et al. (84): Level I = randomized controlled trials or systematic reviews; Level II = cohort studies; Level III = case-control studies; Level IV = case series; Level V = expert opinion. Reported survival benefits should be interpreted within the context of contemporary multimodal oncology, in which surgical outcomes increasingly depend on integration with systemic therapy, multidisciplinary decision-making, and biomarker-informed patient selection, particularly in selected oligometastatic settings (115–117).

## 4 Strategies to mitigate pro-metastatic effects

Evidence that perioperative biological perturbations may contribute to metastatic risk has motivated the development of strategies aimed at attenuating surgery-associated pro-metastatic effects. Approaches under investigation seek to modulate perioperative inflammatory and immune responses, reduce surgical stress and tissue injury, and disrupt tumor-host interactions that support metastatic outgrowth. This section summarizes clinical and translational strategies to mitigate perioperative metastatic risk, with emphasis on pharmacological perioperative interventions, prehabilitation and physiological optimization, perioperative systemic therapy, minimally invasive surgical approaches, and interventions targeting the tumor microenvironment.

### 4.1 Pharmacological perioperative interventions

Recent evidence supports the role of perioperative systemic interventions in limiting surgery-associated metastatic risk (1, 2). Surgical trauma induces a surge in stress hormones and pro-inflammatory cytokines, including IL-6 and TNF-α, which can impair cytotoxic immune responses and promote tumor cell survival, adhesion, and metastatic niche conditioning (2). Contemporary studies further suggest that combining anti-inflammatory agents with perioperative immunomodulation may provide synergistic benefits (1, 59).

Perioperative β-adrenergic blockade combined with non-steroidal anti-inflammatory drugs (NSAIDs) has demonstrated reduced metastatic biomarkers and improved recurrence-free survival in early-phase clinical trials (59, 60). Additionally, targeted perioperative approaches continue to be explored to counteract surgery-induced biological perturbations while balancing safety, immune preservation, and oncologic efficacy. Beyond pharmacological modulation, increasing attention has focused on interventions aimed at strengthening host physiological resilience prior to surgery.

### 4.2 Prehabilitation and perioperative optimization

Increasing attention has focused on prehabilitation as a strategy to improve perioperative physiological resilience and potentially mitigate surgery-associated pro-metastatic effects. Prehabilitation typically combines structured exercise programs, nutritional optimization, psychological support, and risk-factor modification prior to surgery with the aim of enhancing functional reserve and improving postoperative recovery. Emerging clinical evidence indicates that multimodal prehabilitation can improve postoperative functional capacity, reduce severe postoperative complications, shorten hospital stay, and enhance overall recovery following major oncologic surgery (49, 50, 61–63).

Exercise-based prehabilitation may be particularly relevant in the context of perioperative metastatic risk because physical activity exerts important immunomodulatory and anti-inflammatory effects. Experimental and clinical studies suggest that exercise can enhance NK-cell mobilization and cytotoxic activity, improve T-cell function, reduce systemic inflammation, and modulate stress-related neuroendocrine signaling (64, 65). Exercise has additionally been associated with reduced levels of pro-inflammatory cytokines and improved metabolic regulation, potentially counteracting some of the perioperative inflammatory and immunometabolic alterations associated with tumor progression (65, 66).

Nutritional optimization also represents a critical component of perioperative resilience. Malnutrition, sarcopenia, hypoalbuminemia, and metabolic dysfunction are associated with impaired wound healing, postoperative infections, prolonged recovery, and poorer oncologic outcomes (49, 63, 67). Preoperative nutritional support may therefore help preserve immune competence and reduce the severity of postoperative inflammatory and catabolic responses. Psychological support and stress-reduction strategies may further contribute to perioperative immune preservation by attenuating activation of the hypothalamic–pituitary–adrenal axis and sympathetic stress responses that suppress cell-mediated immunity (1).

Although direct evidence linking prehabilitation to reduced metastatic recurrence remains limited, growing evidence supports its role in improving perioperative physiological reserve and reducing postoperative morbidity. These benefits may have favorable implications for long-term oncologic outcomes through enhancement of immune recovery, reduction of systemic inflammatory burden, and improved tolerance to multimodal cancer therapy (50, 63, 67). Future prospective studies integrating immune profiling, inflammatory biomarkers, and recurrence outcomes will be important to clarify whether perioperative prehabilitation can directly modulate surgery-associated metastatic vulnerability.

While prehabilitation focuses on optimizing host resilience before surgery, complementary perioperative strategies have also aimed to directly target residual tumor cells and micrometastatic disease during the surgical window. One such approach is perioperative chemotherapy, which seeks to integrate systemic anticancer treatment with surgical management to reduce postoperative recurrence risk.

### 4.3 Perioperative chemotherapy

Perioperative chemotherapy involves the administration of systemic anticancer agents immediately before or after surgical tumor resection. This strategy aims to eliminate residual tumor cells and micrometastatic disease that may persist or be disseminated during surgical manipulation of the primary tumor (68, 69). Increasing evidence indicates that the perioperative period represents a critical window in cancer progression, during which surgical stress, inflammation, and immunosuppression may promote tumor cell dissemination and metastatic establishment (3, 5). Accordingly, perioperative chemotherapy has been investigated as a strategy to eradicate micrometastatic disease and reduce recurrence risk, with randomized trials and meta-analyses demonstrating improved outcomes in selected malignancies (68, 69).

The rationale for perioperative chemotherapy is based on the premise that tumor cells released during surgical manipulation enter the circulation and contribute to metastatic dissemination (70). Early systemic therapy during this window may help limit the establishment of metastatic niches. In addition, perioperative interventions may counteract the pro-metastatic effects of surgical stress, including the release of growth factors, inflammatory mediators, and angiogenic signals that promote tumor cell survival and colonization (5).

Clinical and translational studies have evaluated perioperative chemotherapy in malignancies where systemic therapy is part of multimodal management. In colorectal cancer, perioperative chemotherapy improves recurrence-free survival and may eradicate micrometastatic disease (69). Targeted therapies have also been explored in the perioperative setting, although results remain heterogeneous. In parallel, biomarker-driven approaches, including circulating tumor cell analysis, have demonstrated potential for detecting occult metastases and refining perioperative risk assessment (71).

Despite these potential benefits, perioperative chemotherapy presents important clinical challenges. Cytotoxic agents may impair wound healing, increase infection risk, or exacerbate perioperative complications if administered too close to surgery (5). Careful optimization of drug selection, dosing, and timing is therefore essential. Advances in perioperative care and improved patient stratification have enhanced the feasibility of integrating systemic therapy into the surgical window while minimizing toxicity (69). Biomarker-based approaches may further aid preoperative risk stratification and identification of patients at higher risk of occult metastatic disease (71).

Overall, perioperative chemotherapy represents a promising adjunct strategy to mitigate the metastatic risks associated with surgical tumor resection. However, further well-designed clinical trials are required to determine optimal therapeutic regimens, timing, and patient selection (68).

Although perioperative chemotherapy aims to reduce metastatic risk through systemic eradication of residual and micrometastatic disease, modification of the surgical approach itself has also emerged as an important strategy for influencing perioperative biology. In this context, minimally invasive surgery has gained increasing attention for its potential to reduce tissue trauma, attenuate inflammatory and neuroendocrine responses, preserve immune function, and thereby mitigate surgery-associated conditions that may favor metastatic progression.

### 4.4 Minimally invasive surgery

Beyond reduced inflammatory responses, minimally invasive surgery (MIS) may influence metastatic risk by limiting tumor cell dissemination during surgical handling. Comparative studies have reported lower perioperative release of CTCs following laparoscopic versus open oncological procedures, particularly in colorectal and gynaecological cancers (72). Reduced tumor manipulation, smaller incisions, and decreased exposure of tumor tissue to the systemic circulation are proposed mechanisms underlying these observations (73). As the presence of CTCs is associated with metastatic potential, perioperative reductions in tumor cell shedding may represent an important advantage of minimally invasive surgical approaches (1, 72).

Despite these benefits, surgical stress induces a marked suppression of immune competence in the immediate postoperative period, including impaired function of NK-cells, which has been observed following both open and minimally invasive surgical approaches (74). Activation of the hypothalamic–pituitary–adrenal axis and sympathetic nervous system during surgery leads to increased levels of catecholamines and glucocorticoids, which suppress cytotoxic immune responses essential for the clearance of disseminated tumor cells (1, 4). These findings suggest that while MIS may reduce mechanical dissemination of tumor cells, it does not fully mitigate surgery-induced systemic immune suppression.

The interaction between reduced tumor cell dissemination and transient postoperative immune dysfunction highlights the complexity of metastatic risk during the perioperative period. Historical and contemporary analyses demonstrate that surgical removal of the primary tumor can disrupt tumor–host equilibrium, potentially awakening dormant micrometastases through inflammatory and angiogenic signaling (1, 3). Minimally invasive surgery may attenuate but not eliminate these systemic effects, underscoring the need to consider both local surgical factors and host biological responses when evaluating metastatic risk.

Advances in robotic-assisted surgery further enhance the technical advantages of minimally invasive approaches (75, 76). Improved visualization, enhanced dexterity, and greater precision may reduce inadvertent tumor compression, vascular injury, and surrounding tissue trauma (73, 75). These features are particularly relevant in anatomically complex oncological resections, where meticulous dissection may limit tumor disruption and reduce perioperative complications (76, 77). Importantly, robotic-assisted surgery has been associated with reduced blood loss and blood transfusion, shorter hospital stays, and faster recovery, all of which may influence postoperative immune recovery (75–77).

Nevertheless, long-term oncological and metastatic outcomes following minimally invasive and robotic-assisted surgery remain an area of active investigation. While oncological equivalence to open surgery has been demonstrated for several cancer types, definitive evidence linking MIS to reduced metastatic recurrence is still emerging (73). Current literature therefore supports a cautious conclusion that minimally invasive surgical techniques reduce perioperative morbidity and tumor manipulation, as their protective effects against metastasis are likely context-dependent and influenced by tumor biology, host immune status, and perioperative systemic factors (60, 73).

Taken together, the available evidence suggests that MIS may contribute to narrowing the perioperative window of metastatic vulnerability when integrated within a broader perioperative strategy. Reductions in tumor cell dissemination and surgical trauma must be considered alongside systemic responses such as immune suppression and inflammatory signaling, which persist regardless of surgical approach (1, 74). Consequently, the greatest benefit of MIS may be realized when combined with perioperative interventions targeting immune preservation and stress modulation, rather than as a standalone strategy to prevent metastatic progression.

Although MIS may mitigate some of the adverse biological consequences of surgical trauma, surgical technique alone is unlikely to fully prevent perioperative conditions that support metastatic progression. This has prompted growing interest in directly targeting the tumor microenvironment and tumor-host interactions during the perioperative period as complementary strategies to suppress pro-metastatic signaling and improve long-term oncologic outcomes.

### 4.5 Targeting the tumor microenvironment

The perioperative period represents a biologically critical window during which surgical stress can profoundly alter the tumor microenvironment (TME) in ways that favor metastatic progression. Surgical resection triggers neuroendocrine and inflammatory responses that promote angiogenesis, immune suppression, and the activation of dormant micrometastases (2, 3). These effects include increased levels of pro-angiogenic factors such as vascular endothelial growth factor (VEGF), reduced concentrations of endogenous angiogenesis inhibitors, and suppression of cell-mediated immunity, collectively creating conditions conducive to metastatic outgrowth (1, 60). Targeting these perioperative microenvironmental changes has therefore emerged as a promising therapeutic strategy.

Experimental and clinical evidence indicates that perioperative pharmacological modulation of stress-induced pathways can attenuate pro-metastatic signaling. Inhibition of catecholamine and prostaglandin pathways reduce inflammatory cytokine release, preserve natural killer cell function, and suppresses molecular programs associated with EMT and tumor invasion (59, 74). These findings suggest that short-term interventions during the perioperative window may have beneficial effects on long-term oncological outcomes.

Neoadjuvant and perioperative immunotherapy has demonstrated significant potential to reprogram the tumor microenvironment and prime durable systemic antitumor immune responses (72, 78). Preclinical studies suggest that administration of immune checkpoint blockade prior to tumor resection may generate stronger and more durable systemic antitumor immunity compared with postoperative treatment alone, partly because the intact primary tumor serves as a continuous source of tumor antigens capable of priming and expanding tumor-specific T-cell populations (72, 78). In this setting, neoadjuvant immunotherapy may enhance activation of cytotoxic CD8^+^ T cells, increase immune memory formation, and improve eradication of residual micrometastatic disease following surgery. Clinical studies have further demonstrated that neoadjuvant immune checkpoint blockade can enhance pathological responses and systemic antitumor immunity (79).

Clinical translation of this concept has been supported by several landmark trials in resectable non-small-cell lung cancer (NSCLC). The phase III CheckMate 816 trial demonstrated that neoadjuvant nivolumab combined with platinum-based chemotherapy significantly improved pathological complete response and event-free survival compared with chemotherapy alone, without compromising surgical feasibility (80). Similarly, the phase III Neotorch trial reported improved event-free survival and major pathological response rates with perioperative toripalimab plus chemotherapy in patients with resectable NSCLC (81). Collectively, these studies support the concept that neoadjuvant chemoimmunotherapy can induce more effective systemic antitumor immune responses while simultaneously reducing tumor burden prior to surgery.

Beyond NSCLC, perioperative immunotherapy is increasingly being investigated across multiple solid tumors as part of broader strategies aimed at modifying perioperative tumor–host interactions. However, despite encouraging early results, important challenges remain regarding optimal patient selection, biomarker identification, treatment timing, and management of perioperative immune-related adverse events. Further biomarker-driven and cancer-specific trials will be necessary to define how perioperative immunomodulation can be integrated into precision oncologic surgery.

**Figure 1:**
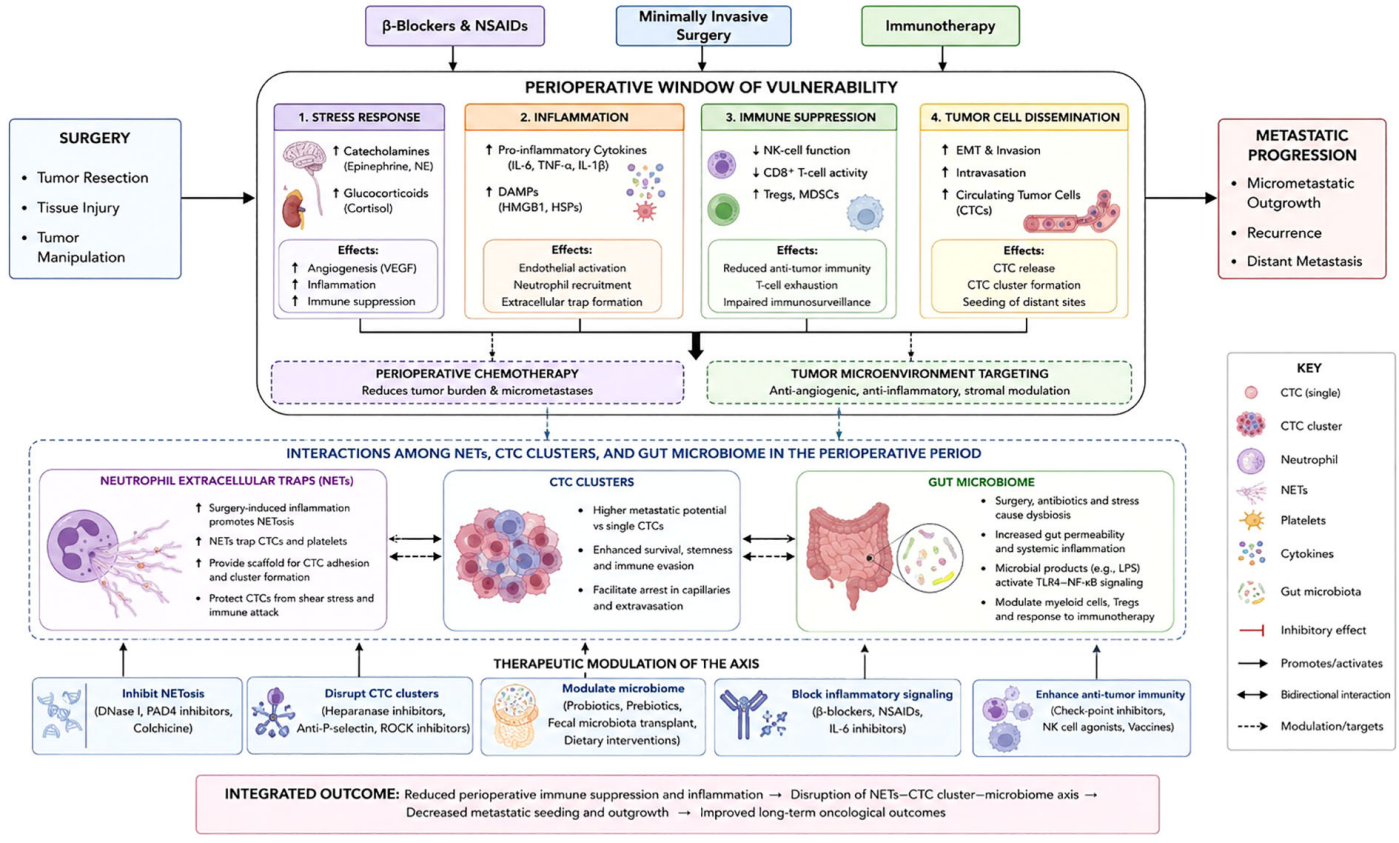
Surgery-associated mechanisms influencing metastatic progression during the perioperative window of vulnerability and potential perioperative intervention targets. Surgical tumor resection induces neuroendocrine stress responses, inflammation, immune suppression, and tumor cell dissemination, collectively creating a pro-metastatic microenvironment. Perioperative inflammatory signaling promotes neutrophil extracellular trap (NET) formation, circulating tumor cell (CTC) survival and clustering, endothelial adhesion, and metastatic niche establishment. Surgery-associated gut microbiome dysbiosis may further contribute to systemic inflammation, immune dysregulation, and tumor-promoting signaling. The figure additionally highlights therapeutic strategies aimed at modulating these pathways, including β-blockers and NSAIDs, perioperative chemotherapy, minimally invasive surgery, immunotherapy, microbiome modulation, and tumor microenvironment targeting, with the goal of reducing postoperative metastatic progression and recurrence.

The interplay between surgery-induced biological responses and potential perioperative intervention targets is summarized in **Figure 1**, highlighting key mechanisms driving metastatic progression and opportunities for therapeutic modulation during the perioperative window of vulnerability.

## 5 Discussion

Accumulating evidence reviewed here supports the concept that surgical intervention can influence metastatic progression through complex and context-dependent biological mechanisms. Experimental and translational studies indicate that perioperative inflammation, immune suppression, neuroendocrine activation, wound-healing responses, and evolving mechanisms involving NETs, CTC clusters, and tumor microenvironment remodeling may promote tumor cell survival, dissemination, immune evasion, and phenotypic plasticity. Clinical observations, including recurrence hazard patterns, perioperative immune dysfunction, and persistence of CTCs, are broadly consistent with these mechanistic frameworks, although direct causal relationships in humans remain difficult to establish.

Importantly, much of the available clinical evidence linking surgery to metastatic risk is associative rather than mechanistic. Recurrence timing analyses, while reproducible across tumor types such as breast and colorectal cancer, do not directly demonstrate surgery-induced dissemination or dormancy escape at the level of individual tumor cells. Similarly, perioperative increases in CTCs and immune suppression are transient and heterogeneous across patients, reflecting variability in tumor biology, disease burden, surgical extent, and host immune competence. These limitations highlight a critical knowledge gap: the lack of real-time, patient-specific evidence linking perioperative biological changes to subsequent metastatic outgrowth. As such, caution is warranted when extrapolating preclinical findings to clinical practice, and surgery remains indispensable for curative intent.

Nonetheless, the convergence of mechanistic and clinical data supports the perioperative period as a critical window of biological vulnerability and therapeutic opportunity. Emerging perioperative interventions including β-adrenergic blockade, anti-inflammatory strategies, immunomodulation, neoadjuvant and perioperative immunotherapy, prehabilitation and physiological optimization, MIS approaches, and TME targeted strategies offer promising avenues to attenuate surgery-associated pro-metastatic signaling. However, their clinical translation remains limited by a lack of adequately powered, biomarker-driven trials and insufficient understanding of which patients are most likely to benefit. The greatest impact of these strategies is therefore likely to be achieved through precise patient stratification and integration within multidisciplinary treatment frameworks.

Several key knowledge gaps must be addressed to advance this field. First, there is a need for robust, validated biomarkers capable of identifying patients at high risk of perioperative metastatic progression. These may include CTCs, circulating tumor DNA, immune profiling (e.g., NK-cell function), inflammatory signatures, and emerging biomarkers reflecting perioperative physiological reserve and tumor-host interactions. However, their predictive value, temporal dynamics, and clinical utility remain to be fully established.

Second, the optimal timing, sequencing, and combination of perioperative interventions remain poorly defined. Critical questions include whether interventions should be administered preoperatively, intraoperatively, or postoperatively; how long such interventions should be maintained; and how they should be combined with standard systemic therapies such as chemotherapy, targeted therapy, or immunotherapy. Addressing these questions will require carefully designed translational studies integrated within clinical trials.

Third, there is a need for prospective, mechanism-informed clinical trials that move beyond surrogate endpoints to evaluate meaningful oncologic outcomes, including metastatic recurrence and long-term survival. Such trials should incorporate longitudinal biomarker sampling to directly link perioperative biological modulation with clinical benefit, thereby bridging the gap between mechanistic insight and patient outcomes.

Fourth, tumor- and patient-specific heterogeneity must be more explicitly addressed. The impact of perioperative biology likely differs across cancer types, stages, and molecular subtypes, as well as across patient populations with varying immune competence and comorbidities. Future studies should therefore prioritize stratified or adaptive trial designs that account for this heterogeneity.

Finally, greater emphasis is needed on integrated perioperative oncology models that combine surgical technique, perioperative physiological optimization, pharmacological modulation, immunological support, and biomarker-informed patient selection. Understanding how minimally invasive approaches, anesthetic techniques, systemic therapies, prehabilitation, and host physiological resilience interact to influence perioperative biology will be critical for developing comprehensive strategies to reduce metastatic risk and improve long-term oncologic outcomes.

To enhance clinical applicability, key practice-oriented considerations and representative supporting clinical evidence for perioperative management are summarized in **Table 3**, providing an evidence-informed framework to support perioperative strategies aimed at mitigating metastatic risk.

**Table 3:** Clinical implications, supporting evidence, and practice pearls for perioperative management to mitigate metastatic risk.

Domain	Representative clinical evidence (recent meta-analyses/trials)	Practice pearl	Clinical implication
Perioperative biology	Hiller et al. (2); Ben-Eliyahu (4)	Recognize the perioperative period as a biologically vulnerable window for metastatic progression	Heightened awareness may inform perioperative planning and multidisciplinary decision-making
Surgical approach	Tai and Auer (9); Dubowitz et al. (48)	Minimize surgical stress and tissue trauma, including use of minimally invasive techniques where appropriate	May attenuate perioperative inflammatory and immunological perturbations associated with metastatic progression
Tumor manipulation	Tohme et al. (5)	Limit excessive tumor handling during resection	May decrease intraoperative release of circulating tumor cells
Perioperative pharmacology	Shaashua et al. (59); Onuma et al. (74)	Consider perioperative use of β-blockers and NSAIDs in selected patients (investigational)	Potential to attenuate stress and inflammatory pathways associated with metastasis
Anesthetic technique	Jin et al. (46); Choi and Hwang (45); Dubowitz et al. (48)	No universal superiority of anesthetic technique has been established; anesthetic selection should be individualized	Possible context-specific effects on perioperative immune function and oncologic outcomes remain under investigation
Systemic therapy timing	Forde et al. (80); Lu et al. (81)	Optimize timing and sequencing of perioperative chemotherapy or immunotherapy	Balance oncologic efficacy with surgical safety and postoperative recovery
Prehabilitation and perioperative optimization	Gillis et al. (118); Barberan-Garcia et al. (61); Minnella et al. (67)	Implement multimodal prehabilitation including exercise, nutrition, and psychological support where feasible	May improve physiological reserve, reduce severe postoperative complications, and support immune recovery
Multidisciplinary care	Torresan et al. (115); Rossi et al. (116)	Integrate perioperative planning within a multidisciplinary team	Enables personalized treatment strategies and coordinated care
Patient selection	Liu et al. (117); Torresan et al. (115)	Stratify patients based on tumor biology, disease stage, treatment context, and overall clinical fitness	Identifies patients most likely to benefit from perioperative interventions
Biomarkers	Tie et al. (119); Reinert et al. (120); Parikh et al. (121)	Use emerging biomarkers (e.g., circulating tumor cells, ctDNA) cautiously for risk assessment	Biomarker-guided stratification may improve recurrence monitoring and perioperative risk assessment but is not yet standard of care
Postoperative care	Bakos et al. (12); Ben-Eliyahu (4)	Monitor and support immune recovery in the early postoperative period	May reduce susceptibility to metastatic outgrowth and improve postoperative resilience

Representative clinical evidence includes selected randomized trials, meta-analyses, and translational studies relevant to perioperative oncology. Recommendations remain investigational in several domains and should not be interpreted as formal clinical practice guidelines.

Notably, recent advances in perioperative oncology, immunotherapy, biomarker development, and multimodal treatment frameworks continue to reshape understanding of surgery-associated metastatic biology, underscoring the importance of maintaining contemporary evidence integration in future translational and clinical studies.

## 6 Conclusion

The relationship between surgery and cancer metastasis is complex and multifactorial, encompassing both tumor-inhibitory and tumor-promoting effects that vary according to tumor biology, surgical context, and host physiological status. While surgical resection remains indispensable for curative cancer treatment, accumulating experimental, translational, and clinical evidence suggests that perioperative inflammation, neuroendocrine activation, immune perturbation, and alterations within the TME may inadvertently create conditions that favor metastatic progression in susceptible contexts. Emerging mechanisms involving CTCs, NETs, and dynamic tumor-host interactions further highlight the biological importance of the perioperative period. Accordingly, the perioperative phase is increasingly recognized as a critical and clinically actionable therapeutic window. Integrating perioperative strategies including systemic therapies, immunomodulation, neoadjuvant and perioperative immunotherapy, MIS approaches, prehabilitation, physiological optimization, and targeted modulation of the TME offers promising opportunities to improve perioperative resilience and mitigate metastatic vulnerability without compromising oncologic efficacy. However, clinical translation remains incomplete, and several proposed perioperative interventions including β-adrenergic blockade, NSAIDs, and anesthetic modulation strategies remain investigational for the prevention of metastatic recurrence and are not yet supported by definitive guideline-based recommendations. Future progress will depend on biomarker-informed patient stratification, multidisciplinary perioperative care models, and rigorously designed mechanism-informed clinical trials that integrate immune profiling, circulating biomarkers, and long-term oncologic outcomes. Such efforts may enable more precise and personalized perioperative interventions aimed at reducing metastatic risk and improving long-term cancer outcomes.
